# The contribution of family physicians in coordinating care and improving access at district hospitals: The False Bay experience, South Africa

**DOI:** 10.4102/phcfm.v13i1.3226

**Published:** 2021-11-18

**Authors:** Liezel Rossouw, Hoosain Lalkhen, Kaashiefah Adamson, Klaus B. von Pressentin

**Affiliations:** 1 Division of Family Medicine, School of Public Health and Family Medicine, Faculty of Health Sciences, University of Cape Town, Cape Town, South Africa; 2 False Bay District Hospital, Metro District Health Services, Western Cape Department of Health, Cape Town, South Africa; 3 Department of Family and Emergency Medicine, Faculty of Medicine and Health Sciences, Stellenbosch University, Cape Town, South Africa

**Keywords:** family physicians, leadership, clinical governance, district health system, care coordination, access to care, teamwork, South Africa

## Abstract

This short report describes three family physicians (FP)-led clinical governance interventions to strengthen the care access and coordination in an urban district hospital in Cape Town, South Africa. The actual experiences and their effects on health services are captured here. The report also describes a range of interventions from enhanced access to timely computer tomographic scans to determine definitive care, to creating a local referral forum between levels of care, which resulted in a renewed appreciation for the scope of services and illness burden managed by the district health system and to the establishment of an onsite echocardiology service at the local district hospital to enhance the identified burden of disease of the local community. Each of these interventions were planned and implemented based on local data in partnership with the team members at the different levels of care. By applying an inclusive and distributed leadership style as informed by care access to scarce resources was better coordinated for the local communities served. The importance of the building trusting relationships between FPs and referral hospital colleagues cannot be overemphasised. Family physicians should be integrated and collaborated in the clinical governance platforms across levels of care. The FP’s roles as primary care consultant and clinical governance leader are pivotal in enhancing service delivery efficiency and in providing quality healthcare.

## Focus of the report

This report from Cape Town, South Africa, describes three examples of how a group of family physicians (FPs) strengthened health services within their sphere of influence, to enhance access to more efficient and appropriate care for their patients. False Bay District Hospital (FBDH) is a public sector, level one hospital situated in Fish Hoek, which is located about 30 km (40-min by road) from the Groote Schuur tertiary referral hospital in the Cape Town centre and serves the Cape Peninsula communities. It has a 76-bed inpatient service, an outpatient service, theatre, a midwife obstetric unit, a 72-h inpatient psychiatric unit, and a 24-h emergency unit. The clinical team consists of two full-time FPs working within a team of medical officers, interns, family medicine registrars (enrolled for postgraduate training in the Division of Family Medicine at the University of Cape Town), rehabilitation and nursing professionals.

## Example 1: Access to and clinical governance of computer tomographic scans

### Contextual description

Historically, all urgent and semi-urgent computer tomographic (CT) scans from FBDH were performed at Groote Schuur Hospital (GSH). False Bay District Hospital patients would wait for four months to access semi-urgent CT scans. This resulted in a delay in the diagnosis and definitive management of patients with cancer. Local data showed that a quarter of deaths from the FBDH drainage area were because of cancer. In March 2019, Victoria Hospital in Wynberg (VHW), a level 2 referral hospital around 20 km (25-min by road) from FBDH, commissioned a daytime CT radiology service. This additional service helped to decrease the waiting time for FBDH patients to 30 days. However, semi-urgent patients presenting at FBDH appeared to be disadvantaged in accessing a timeous CT scan compared with those presenting at higher levels of care (levels 2 and 3).

A meeting between FBDH, GSH and VHW representatives was held on 20 September 2019 to discuss the appropriateness of district hospital CT bookings without an additional clinical assessment at VHW. There seemed to be an assumption that FBDH clinicians were ordering unnecessary CT scans, as it was suggested that FBDH patients transferred for urgent and semi-urgent CT scans required review by the VHW clinicians to confirm the indication. However, VHW was only open for CT scans until 16:00 on weekdays, which would cause an estimated 30-h delay in urgent patients receiving a CT scan. Furthermore, this approach would increase costs and workload for both the emergency medical transport and VHW services.

The FPs suggested that a FP review of all FBDH CT scan requests would ensure appropriate referrals. In other words: would a FP-governed CT scan booking system be able to decrease the waiting time for patients requiring semi-urgent CT scans?

### Intervention: Access and governance of a CT scan service

The FPs conducted a retrospective before and after audit of the referrals for CT scan (Table 1). A list of all the patients referred for a CT scan from FBDH were collated from the electronic radiology picture archiving and communication system (PACS), as well as the emergency unit and ward referral registers. False Bay District Hospital referred 42 patients for CT scans during July 2019, which accounted for 0.5% (*n* = 8600) of all patients seen during this period. Positive CT scan results were recorded in 62% (*n* = 26) of these patients. The positivity rate may be considered too high, indicating likely underdiagnosis because of resource constraints.^1^

**TABLE 1:** Comparison of the audit carried out at False Bay District Hospital before and after the CT scan governance intervention.

Audit date	Before and after intervention comparison			
	Waiting time for semi- urgent CT scan appointment	Total number of patients scanned	Number of inappropriate CT scan referrals	Number of CT scan referrals not iscussed with a FP
**July 2019**				
*N*	30 days	42	6	6
%	-	-	-	33
**February 2020**				
*N*	3 days	38	0	0
%	-	-	-	0

CT, computer tomographic; FP, family physician.

The audit showed that the waiting time for CT scans was dramatically reduced, whilst the number of CT scans remained more or less the same and the number of inappropriate requests was also reduced. All CT scan bookings were deemed appropriate (Table 1). Emergency CT scans could be accessed the same day, admitted patients could access CTs within 2 days and semi-urgent patients within two weeks. No morbidities or adverse outcomes because of delayed diagnoses of non-benign pathologies were reported.

It was concluded that good clinical governance systems were in place at FBDH and that FPs were well positioned to screen and coordinate special investigation usage, which is in line with the views previously expressed by key South African leaders.^2^ Subsequently, the recommendation and agreement with the level 2 and 3 colleagues included that same day or urgent CT scans should continue at GSH, as these represented a small number (8–10 CT scans per month). A dedicated CT scan slot at VHW for FBDH patients was proposed. It was agreed that the bookings will be managed electronically by the FBDH FPs. This was performed via Google booking sheets on smartphone devices. This system ensured that more urgent patients are prioritised and that all referred patients were sent directly to the CT radiology service without the need for an additional level 2 or 3 clinician review unless requested by the FBDH clinicians.

### Conclusion

This intervention decreased the administrative and screening burden on the CT radiology service and reassured the referral hospital managers and specialists that the FBDH referrals were appropriate. The presence of FPs enabled a change in access to special investigations that resulted in decreased cost to the system from multiple clinical assessments of the same patient at different levels of care and additional emergency ambulance transport. Most importantly, the new system negated a delay in patients receiving definitive care.

## Example 2: Creating a local referral forum

### Contextual description

Historically, relationships between different levels of care in the local referral network were strained because of miscommunication and a lack of understanding or appreciation of the FBDH’s contextual realities. The prevailing level 2 and 3 impression was that too many patients were referred to the higher levels of care.

### Intervention

The FPs invited the level 2 VHW specialists to a meeting on 08 October 2020, which aimed to strengthen the relationships between the VHW and FBDH teams. At the meeting, the lead FP presented data of acute patient referrals to VHW. In addition, information was presented on the number of patients treated at FBDH and the scope of services, including additional services implemented under the leadership of the FPs (ultrasound, echocardiogram and gastroscopy services). This meeting concluded with a tour of FBDH.

**FIGURE 1:**
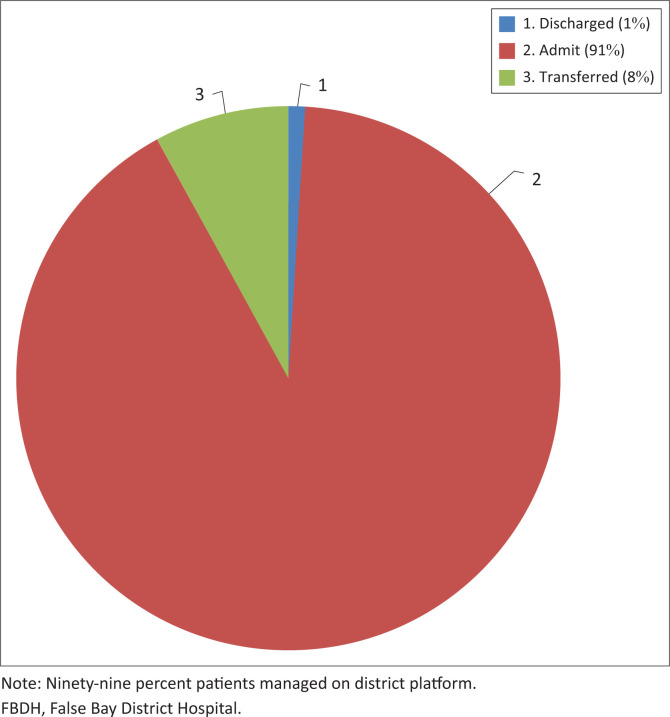
Status of patients seen and managed at False Bay District Hospital during September 2020 (*n* = 5732).

Data from a 2-week audit (17 September 2020 to 01 October 2020) were presented, which showed that 1591 patients were seen at the FBDH emergency centre. Of all acute patients seen in the emergency centre and admitted to FBFH, 48 patients (3% of acute patients seen) required referral to other levels of care (including VHW and GSH). Most patients admitted to FBDH (147 patients; 95%) were managed at the district level. The number of patients referred to VHW was low (17 patients) and these referrals were deemed appropriate (access to resources not available at FBDH, including specialised theatre and high care services). Figure 1 shows the outcomes of all the patients managed in the emergency, inpatient and outpatient services at FBDH during September 2020.

During the October 2020 meeting, a referral forum and an ongoing audit of referrals was proposed, in order to strengthen the understanding of the role and scope of the district hospital service.

### Conclusion

Victoria Hospital in Wynberg specialists were surprised by the large number of patients managed at FBDH, which helped to challenge their assumption that FBDH clinicians transferred majority of the acute patients to VHW. They were impressed by the sound clinical governance processes in place and that FBDH clinicians were able to manage paediatric and adult patients across all specialities in their wards. The audit illustrated how the presence of FPs in the district hospital reduced the need to refer patients to the next level of care.^2^ Subsequent to the meeting, both FPs and medical officers reported an improved referral experience to VHW. Improving coordination of care across the primary–secondary care interface requires building relationships of mutual respect and trust and overcoming ‘professional tribalism’.^3^ One strategy for doing this is the development of forums where clinicians can discuss challenges and solutions.

## Example 3: Creating an onsite echocardiography screening service

### Contextual description

In 2017, FPs at FBDH reviewed the annual mortality data between 2015 and 2017 and found that more than a third of deaths (39%) were cardiac in origin. A cross-sectional audit was conducted in the outpatient department during September 2017, which highlighted a significant problem for cardiac patients in accessing definitive care. Referral letters faxed to cardiology for appointments were accessed on the Clinicom (Electronic Clinical Event Manager) system. In total, 27 cardiology referral letters were reviewed. On average, patients waited for 9-months to be informed of their appointment date and 12-months for the actual appointment.

### Intervention: Leadership and accessibility of care

The FPs presented the data to the head of the GSH cardiology service and proposed a new email referral system. The FPs would screen all referrals, which could significantly reduce the number of referrals. The lead FP at FBDH attended a talk on cardiology at the VHW/FBDH clinical forum and communicated the need for an onsite echocardiology service at FBDH. The cardiologist presenter, Dr Tony Lachman, offered to assist the FP with setting up this service, as a way of transferring knowledge, free of charge. The FBDH cardiology clinic consisted of patients seen and screened by the FP. The clinic was attended by the FP and supported by the outreach cardiologist, Dr Lachman.

The clinic has subsequently managed 60 patients per month over the past 2-years. This resource dramatically reduced unnecessary cardiology referrals to VHW and GSH. Appropriate patients were evaluated and managed at the correct level of care within an optimal time frame. Cardiology appointments at GSH were secured within 30-days.

### Conclusion

The outreach clinic contributed towards more trusting relationships between the levels of care, which resulted in improving referrals and waiting times, as well as access to services at the district hospital. The lead FP acquired the skill to perform telemedicine-assisted echocardiography, which enabled acute care diagnosis and informed cardiac failure therapy plans at the district hospital.^4,5^ This example also illustrates the role that FPs can play in seeing the bigger picture, improving systems and advocating for change.

## Reflections on these three experiences

These three interventions demonstrate clearly how FPs may use their understanding and clinical experience of the local burden of disease, system inequities, as well as available data to advocate for improved patient care access and coordination.^2,6,7^ Most patients are managed on the district platform and decisions around care pathways were designed in a bottom-up approach.^6,8,9,10^ The importance of building trusting relationships between FPs and referral hospital colleagues cannot be overemphasised.^10^ Family physicians should be integrated with and collaborate in the clinical governance platforms across levels of care.^7,10,11^ This can be done by analysing referrals, sharing statistics and setting up inter-hospital referral forums.^2,6^ The FPs role of primary care consultant and clinical governance leader are pivotal in enhancing service delivery efficiency and in providing quality healthcare.^10,11^

Family physicians are ideally positioned to draw on their system and experiential knowledge to identify service needs. The FP-led innovations described here illustrate that it is possible to enhance access to equitable, efficiently coordinated and quality patient-centred care closer to their patients’ homes. This report demonstrates how FPs may realise their leadership and clinical governance roles in a relationship-centred manner. The report also demonstrates the value of employing FPs at district hospitals and whilst this has become the norm in the Western Cape, it is not seen in many other provinces.
