# Erratum: The contribution of family physicians in coordinating care and improving access at district hospitals: The False Bay experience, South Africa

**DOI:** 10.4102/phcfm.v14i1.3353

**Published:** 2022-07-04

**Authors:** Liezel Rossouw, Hoosain Lalkhen, Kaashiefah Adamson, Klaus B. von Pressentin

**Affiliations:** 1 Division of Family Medicine, School of Public Health and Family Medicine, Faculty of Health Sciences, University of Cape Town, Cape Town, South Africa; 2 False Bay District Hospital, Metro District Health Services, Western Cape Department of Health, Cape Town, South Africa; 3 Department of Family and Emergency Medicine, Faculty of Medicine and Health Sciences, Stellenbosch University, Cape Town, South Africa

In the published article, Rossouw L, Lalkhen H, Adamson K, Von Pressentin KB. The contribution of family physicians in coordinating care and improving access at district hospitals: The False Bay experience, South Africa. Afr J Prm Health Care Fam Med. 2021;13(1), a3226. https://doi.org/10.4102/phcfm.v13i1.3226, there was a mistake in the legend for Figure 1 as published. The wrong labels were allocated to the different parts of the pie chart in the legend.

The original incorrect legend for Figure 1:

1. Discharged (1%)
2. Admit (91%)
3. Transferred (8%)

The revised and updated legend for Figure 1:

1. Transferred (1%)
2. Discharged (91%)
3. Admit (8%)

The publisher apologises for this error. The correction does not change the significance of study’s findings or overall interpretation of its results or the scientific conclusions of the article in any way.

